# Dysregulated cholinergic network as a novel biomarker of poor prognostic in patients with head and neck squamous cell carcinoma

**DOI:** 10.1186/s12885-015-1402-y

**Published:** 2015-05-10

**Authors:** Ana Cristina Castillo-González, Susana Nieto-Cerón, Juan Pablo Pelegrín-Hernández, María Fernanda Montenegro, José Antonio Noguera, María Fuensanta López-Moreno, José Neptuno Rodríguez-López, Cecilio J Vidal, Diego Hellín-Meseguer, Juan Cabezas-Herrera

**Affiliations:** 1 Molecular Therapy and Biomarkers Research Group, Clinical Analysis Service, University Hospital Virgen de la Arrixaca, IMIB-Arrixaca, Ctra Madrid-Cartagena s/n, El Palmar, Murcia, 30120 Spain; 2 Otorhinolaryngology Surgical Service, University Hospital Virgen de la Arrixaca IMIB-Arrixaca, Ctra Madrid-Cartagena s/n, El Palmar, Murcia, 30120 Spain; 3 Department of Biochemistry and Molecular Biology A, School of Biology, Regional Campus of International Excellence “Campus Mare Nostrum”, IMIB-University of Murcia, Murcia, 30100 Spain

**Keywords:** Cholinergic system, Non-neuronal compartment, Human airways, Head and neck cancer

## Abstract

**Background:**

In airways, a proliferative effect is played directly by cholinergic agonists through nicotinic and muscarinic receptors activation. How tumors respond to aberrantly activated cholinergic signalling is a key question in smoking-related cancer. This research was addressed to explore a possible link of cholinergic signalling changes with cancer biology.

**Methods:**

Fifty-seven paired pieces of head and neck squamous cell carcinoma (HNSCC) and adjacent non-cancerous tissue (ANCT) were compared for their mRNA levels for ACh-related proteins and ACh-hydrolyzing activity.

**Results:**

The measurement in ANCT of acetylcholinesterase (AChE) and butyrylcholinesterase (BChE) activities (5.416 ± 0.501 mU/mg protein and 6.350 ± 0.599 mU/mg protein, respectively) demonstrated that upper respiratory tract is capable of controlling the availability of ACh. In HNSCC, AChE and BChE activities dropped to 3.584 ± 0.599 mU/mg protein (*p* = 0.002) and 3.965 ± 0.423 mU/mg protein (*p* < 0.001). Moreover, tumours with low AChE activity and high BChE activity were associated with shorter patient overall survival. ANCT and HNSCC differed in mRNA levels for AChE-T, α3, α5, α9 and β2 for nAChR subunits. Tobacco exposure had a great impact on the expression of both AChE-H and AChE-T mRNAs. Unaffected and cancerous pieces contained principal AChE dimers and BChE tetramers. The lack of nerve-born PRiMA-linked AChE agreed with pathological findings on nerve terminal remodelling and loss in HNSCC.

**Conclusions:**

Our results suggest that the low AChE activity in HNSCC can be used to predict survival in patients with head and neck cancer. So, the ChE activity level can be used as a reliable prognostic marker.

**Electronic supplementary material:**

The online version of this article (doi:10.1186/s12885-015-1402-y) contains supplementary material, which is available to authorized users.

## Background

Head and neck carcinomas arise in the mucosal layer of the upper aerodigestive tract (oral cavity, oropharynx, hypopharynx, and larynx). Nearly 90 % of head and neck carcinomas are assigned to squamous cell carcinoma (HNSCC) and with over 600,000 new cases worldwide each year, head and neck neoplasia is the sixth most frequent cancer [1, 2]. Patients with HNSCC at early stage can be cured with aggressive multimodal therapy (surgery, radiation, and/or chemotherapy). Unfortunately, no treatment is still available to reach fully satisfactory achieves, and, therefore, the mortality rate of HNSCC patients remains high [3]. Novel and reliable biomarkers for distinguishing patients with poor prognosis or great risk of early recurrence, and for using personalized therapies are still awaited given to uncertainty in clinical evolution of HNSCC using current staging criteria.

Increasing evidence points out that several non-neural cell types are capable of expressing the range of proteins that form a non-neuronal cholinergic system i.e. the ACh-synthesizing enzyme choline acetyltransferase (ChAT), nicotinic (nAChR) and muscarinic (mAChR) receptors, and the ACh-hydrolyzing enzymes acetyl- (AChE) and butyrylcholinesterase (BChE) (reviewed in reference [4]). Its great catalytic efficiency allows AChE working as an ideal molecular machine for controlling and stopping ACh-elicited actions [5]. On the other hand, most tissues and body fluids contain a second ChE named BChE. Despite the lower catalytic efficiency of BChE than AChE at the time to hydrolyze acetylcholine, BChE contributes to ACh homeostasis as judged by its role in AChE-null mice [6, 7]. The catalytic action of AChE and BChE ensures rapid withdrawal of ACh, which, otherwise, may lead to cholinergic over-activation.

In the context of a cell type-specific cholinergic phenotype, it is worth noting the results that demonstrate that the human respiratory tract epithelium possesses a non-neuronal cholinergic system engaged in controlling the level of ACh. It seems that this epithelial cholinergic system operates actively to regulate auto/paracrine actions and by this means controls reliably basic cell functions [8]. The cell proliferation effects arising from cholinergic over-activation, via endogenous ACh or nicotine-derived carcinogens, gain significance when considering the susceptibility to lung cancer that confers AChR disorders [9], the nicotine-guided shift in the expression pattern of AChR to proliferating/migrating cell phenotypes [10], and the promising therapies based in the blockade or attenuation of cholinergic signalling [11–13].

The information relative to AChE and BChE involvement in cell proliferation and differentiation [14] makes it possible that ChEs take part in tumour development. In support of this idea are: 1) the frequent aberrations in the AChE gene and the structural changes in AChE proteins observed in tumours of diverse origin [8, 15–19]; 2) the expression of AChE during and after apoptosis induction with different stimuli [20–22]; and 3) the profitable use as a prognostic predictor for liver carcinoma of AChE and its profitable effects through suppression of cell growth and enhancement of chemosensitization [23].

The contribution of cholinergic signalling to cancer onset and growth [24] and our previous reports showing that neoplastic transformation alters the level of AChE and/or BChE activities and the content of ChE-mRNAs in human breast, lymph node, intestine, lung, kidney and prostate [8, 18, 25, 26] prompted us to compare unaffected tissue samples and head and neck tumours for possible changes in the expression of AChE and BChE, which would alter the availability of ACh, and to test the usefulness of the changes in ChE expression as reliable diagnostic or prognostic markers.

## Methods

### Patients and samples

A total of 57 human malignant primary carcinomas (HNSCC) and their adjacent noncancerous tissues (ANCT) taken in the surgery act made at *Virgen de la Arrixaca* Clinical University Hospital in Murcia (Spain) from 2007 to 2011 were included in the current study. Fresh specimens were divided into sections and stored at − 80 °C until use. The TNM classification of HNSCC specimens was made according to the UICC:TNM Classification of Malignant Tumours. The study approval and the consent procedure were obtained from the Institutional Ethic Committee of our Hospital. All patients gave their consent after being appropriately informed.

### Extraction and assay of cholinesterases

Cholinesterases were extracted from surgical ANCT and HNSCC pieces by homogenization (5 % w/v) with Tris-saline buffer (TSB; 1 M NaCl, 50 mM MgCl_2_, 3 mM EDTA, 10 mM Tris, pH 7.0) supplemented with 1 % Brij 96 and a fresh mixture of proteinase inhibitors (0.1 mg/ml soybean trypsin inhibitor, 1 mg/ml bacitracine, 0.0022 TIU/ml aprotinin, 10 mg/ml pepstatin A and 20 mg/ml leupeptin). After centrifugation at 30,000 rpm, 1 h at 4 °C, in a 70Ti Beckman rotor (Palo Alto, CA, USA), the supernatant with AChE and BChE was saved. For assays with fluorochrome-tagged physostigmine (Ph-F) proteinase inhibitors were not added to the extraction buffer.

Cholinesterase activity was determined as earlier [8] and protein concentration by BioRad Protein Assay with bovine serum albumin as the standard.

### Sedimentation analysis

Possible differences between ANCT and HNSCC in the molecular distribution of AChE and BChE were tested by sedimentation analysis in sucrose gradients as reported before [8]. Briefly, samples and sedimentation markers (bovine liver catalase and intestine alkaline phosphatase) were layered on the top of centrifuge tubes containing 5 - 20 % sucrose gradients, in the presence of 0.5 % w/v Brij 96 detergent. The gradient tubes were centrifuged at 35,000 rpm in a SW41Ti rotor in a Beckman L–80 OPTIMA XP Ultracentrifuge (Fullerton, CA, USA), 18 h at 4 °C. After centrifugation, fractions of 250 μl were collected from the tube bottom and assayed for AChE and BChE activities and enzyme markers.

### mRNA Isolation and real-time PCR

Differences between ANCT and HNSCC specimens in the expression level of cholinergic components were studied by RT-PCR. For this, mRNA was extracted from tissues using the Chemagic mRNA Direct Kit (Chemagen), and reversed transcribed into cDNA by random priming (GeneAmp RNA-PCR kit, Applied Biosystems). A LightCycler thermocycler (Roche Molecular Biochemicals, Mannheim, Germany) was used for RT-PCR. Pairs of primers were designed for quantitative PCR targeting of the 3’-alternative AChE mRNAs (R, H, or T) and the transcripts for BChE, choline acetyltransferase (ChAT), proline-rich membrane anchor (PRIMA), nAChR subunit genes α3, α5, α7, α9, β2, and β4; and mAChR subunit genes M2 and M3. The transcripts for β-actin and GAPDH were used for internal normalization. Reaction conditions were validated separately for each pair of primers, with single peak of dissociation curves produced in each run of reaction. The sequence and position of the primers, as well as the size of the PCR products, are provided in Additional file 1: Material. The buffered medium contained 5 μL of variable dilutions of cDNA, 0.3 μM specific primers, and a volume of PCR master mix to complete 20 μL. Reactions comprised a first step of 30 sec min at 95 °C, followed by 40 cycles of 10 seconds to 95 °C, 10 seconds at 60 °C, 15 seconds at 72 °C. A final dissociation stage allowed us to study the melting curves. The relative content of cDNAs, with respect to β-actin cDNA, was determined by the second derivative method with kinetic PCR efficiency correction. PCR products were separated in 2 % agarose gels and visualized with GelRed Nucleic Acid Gel Stain (Biotium) to check that their lengths coincided with the expected size. Negative controls (without reverse transcriptase) for each primer pair were also made.

### Western blotting

AChE subunits of ANCT and HNSCC were resolved by reducing SDS-PAGE [27] in 12.5 % polyacrilamide-gel slabs. Proteins were electro-transferred to PVDF membranes, blocked with 5 % non-fat dried milk and incubated with the N19 anti-AChE antisera (Santa Cruz). Since N19 antibodies are produced against the N-terminal peptide of human AChE, they should react with the full set of AChE variants. Labelled proteins were revealed using suitable horseradish peroxidase-conjugated antibodies and the Pierce ECL2 Western blotting substrate (Thermo Scientific). The size of AChE subunits was estimated using appropriate protein standards Full Range Rainbow Molecular Weight Markers (GE Healthcare), and the intensity of the protein bands was quantified using GelPro Analyzer Software (version 3.1; Media Cybernetic). β-actin was used as a loading control.

In addition, the use of fluorescein-tagged physostigmine (Ph-F) allowed us a direct observation of the resolved AChE subunits. For this, protein extracts from non-cancerous and cancerous pieces were adjusted to 1 mg/ml in Tris buffer, and treated with 2 μM Ph-F, 30 min at room temperature. Afterwards, the reaction was quenched by adding its volume of reducing electrophoresis sample buffer. Proteins were separated by SDS/PAGE in 4–12 % polyacrylamide slabs, and visualized in-gel with a GE Healthcare Typhoon™ fluorescence scanner.

### Statistical analysis

The results are given as a mean ± SEM. Numeric data were analyzed for statistical significance using Mann-Whitney test. Statistical significance for mean values was set-up at p < 0.05. Kaplan-Meier curves were constructed to assess disease-free (DFS) or overall (OS) survival. The starting point for survival studies was the date of surgical act and the final point was the manifestation of either local recurrence or distant metastatic dissemination (DFS), or death (OS). Differences between groups were analysed using the log-rank test for equality of survivor. A difference of p < 0.05 was considered to be statistically significant. Data were analyzed using the SPSS software, version 15.0 (SPSS Inc., Chicago, IL).

## Results

### Characteristics of patients

Fifty-seven patients participated in this research (Table 1). They were grouped according to sex, age, tobacco exposure, alcohol consumption, anatomical tumour location, differentiation grade (well versus poor and moderately differentiated), clinical stage (I and II versus III and IV), and lymph node affectation (N0 versus N+).

**Table 1:** Summary of demographic characteristics of HNCSS patients and acetyl- and butyrylcholinesterase activity in upper respiratory epithelium

		AChE activity (mU/mg protein)			BChE activity (mU/mg protein)		
Samples	N	ANCT	Tumour	*P*-value	ANCT	Tumour	*P*-value
**Total**	57	5.416 ± 0.501	3.584 ± 0.633	**0.002**	6.350 ± 0.599	3.965 ± 0.423	**<0.001**
**Gender**							
Male	53	5.094 ± 0.473	3.525 ± 0.674	**0.003**	6.278 ± 0.638	3.952 ± 0.450	**0.001**
Female	4	9.680 ± 2.962	4.363 ± 1.395	0.273	5.730 ± 1.303	3.142 ± 0.843	0.068
**Age**							
<60	18	6.175 ± 0.929	4.553 ± 1.390	0.145	7.007 ± 1.311	4.572 ± 0.808	0.093
>60	39	5.066 ± 0.593	3.136 ± 0.669	**0.005**	5.891 ± 0.648	3.825 ± 0.495	**0.002**
**Tobacco**							
Non-smoker	5	4.978 ± 1.556	3.135 ± 1.820	0.225	3.891 ± 0.876	3.657 ± 0.922	0.893
Smoker	46	5.145 ± 0.510	3.721 ± 0.741	**0.017**	6.852 ± 0.716	3.872 ± 0.431	**<0.001**
**Alcohol**							
No	17	5.219 ± 0.621	3.524 ± 0.695	0.290	5.807 ± 1.034	4.701 ± 0.645	0.650
Yes	16	5.921 ± 0.822	3.737 ± 1.425	**0.034**	6.491 ± 0.745	3.746 ± 0.445	**<0.001**
**Location**							
Glottic	29	4.736 ± 0.663	3.467 ± 0.902	**0.003**	5.978 ± 0.794	3.612 ± 0.697	**0.004**
Supraglottic	21	5.200 ± 0.607	4.423 ± 1.201	**0.017**	7.593 ± 1.205	4.299 ± 0.603	**0.007**
Other	7	8.374 ± 1.699	2.699 ± 0.692	0.052	4.544 ± 0.530	4.362 ± 0.843	1.000
**Differentiation**							
Well	17	5.279 ± 0.828	4.057 ± 1.473	0.227	5.013 ± 0.806	3.771 ± 0.828	0.193
Moderate/Poor	32	5.123 ± 0.637	3.631 ± 0.807	**0.030**	7.684 ± 0.947	4.334 ± 0.622	**0.002**
**Clinical Stage**							
Stage I + II	19	4.660 ± 0.717	2.638 ± 0.714	0.059	6.118 ± 0.849	4.046 ± 0.858	0.058
Stage III + IV	36	5.319 ± 0.598	4.167 ± 0.921	**0.033**	6.568 ± 0.829	3.951 ± 0.504	**0.002**
**Nodal Status**							
N0	30	4.780 ± 0.530	3.231 ± 0.754	0.077	6.438 ± 0.857	3.624 ± 0.534	**0.003**
N+	19	5.301 ± 0.943	3.432 ± 1.335	**0.032**	6.644 ± 1.006	4.878 ± 0.838	**0.039**
**T Stage**							
T1	4	4.707 ± 1.638	1.405 ± 0.670	0.068	4.456 ± 0.583	2.228 ± 0.367	0.068
T2	16	4.485 ± 0.790	2.893 ± 0.822	0.179	7.293 ± 1.302	4.265 ± 0.952	0.063
T3	24	5.727 ± 1.178	4.600 ± 1.562	0.382	6.522 ± 1.681	3.815 ± 0.559	0.110
T4	11	5.224 ± 0.692	4.019 ± 1.214	**0.031**	6.085 ± 0.853	4.331 ± 0.756	**0.028**

The age of patients ranged 24–89 years, with mean ± SD of 66.55 ± 11.42. Most patients were male (93.3 %) as well as current or former smokers (79.8 %). The prevalent tumour location was the larynx (49/57; 85.95 %), with carcinomas distributed between the glottis (28/57; 49.12 %) and supraglottis areas (21/57; 36.84 %). A few HNSCC were located in the hypopharynx (2/57; 3.51 %), oral cavity (4/57; 7.02 %) and paranasal sinus (1/57; 1.75 %) (Table 1). Among the tumours tested, 36/57 (63.16 %) were at late stage (III and IV) and 19/57 (33.33 %) at early stage (I and II). The percentages of well, moderately, and poorly differentiated tumours were 31.58 % (18/57), 40.35 % (23/57), and 33.33 % (19/57), respectively. The analysis of correlation of demographic and pathological parameters with outcome of HNSCC patients is showed in Table 2.

**Table 2:** Correlation of variables with outcome in head and neck carcinomas

Variable	Overall survival (mean survival time in months)	p-value
Sex		
Male	47.24	0.847
Female	48.00	
Age		
<60	56.00	0.046
≥60	43.73	
Smoking		
No	31.38	0.907
Yes	47.96	
Alcohol intake		
No	49.41	0.246
Yes	40.09	
Site of primary tumor		
Glottic	50.07	0.751
Supraglottic	47.10	
Other	43.17	
Differentiation grade		
Well	47.79	0.792
Moderate/Poor	49.38	
Clinical stage		
Stage I + II	55.94	0.034
Stage III + IV	42,34	
Nodal Status		
N0	52.66	0.033
N+	38.95	

### Both AChE and BChE activities were decreased in head and neck carcinomas

The observation in non-neural human tissues of cholinergic components [28] prompted us to examine their expression levels in human upper respiratory tract epithelium. So, taking into account the importance of ChEs for regulating ACh levels, and therefore, for controlling the intensity and duration of cholinergic signals, ChE activity levels in ANCT and HNSCC pieces were compared. The observation in ANCT of AChE and BChE activities (5.416 ± 0.501 mU/mg protein and 6.350 ± 0.599 mU/mg protein) demonstrated that upper respiratory tract is able to regulate the availability of ACh. In HNSCC, AChE and BChE activities dropped to 3.584 ± 0.633 mU/mg (*p* = 0.002) and 3.965 ± 0.423 mU/mg (*p <* 0.001), respectively (Table 1).

A possible pathological significance for the changing ChE activity was examined by comparing AChE and BChE activities in tumours and their clinico-pathological parameters. The results showed that AChE activity in HNSCC was significantly lower relative to ANCT in the smokers group (p = 0.017), alcohol drinking group (p = 0.034), moderate and poor differentiation grade (p = 0.030), clinical stage III + IV group (p = 0.033), and lymph node-positive group (p = 0.032) (Table 1). No association between AChE activity and HNSCC aggressiveness was observed. Since it has been reported that serum AChE levels go up with age [29], tissue ChE activities were tested in association with age. No significant association between AChE activity and age was found (Spearman's rank correlation coefficient = 0.059, p = 0.663; Pearson's correlation coefficient = 0.053, P = 0.695).

As regards BChE activity, its level was also found significantly decreased in cancerous pieces (Table 1). Lower BChE activity levels in tumours with respect to their ANCT correlated with smoking (p < 0.001), alcohol consumption (p < 0.001), poorer differentiation grade (p = 0.002) and advanced clinical stage (p = 0.002).

AChE and BChE activity was measured in serum from cancer patients and in age-matched control group. Serum in all HNSCC patients contained significant level of both AChE and BChE activity. Values of both AChE and BChE were in the normal range thus it can be excluded that differences in cholinesterase levels exist due to genomic changes. Serum AChE activity was 0.63 ± 0.08 mU/ml and BChE activity was 140.47 ± 7.24 mU/ml. However, the results indicated that both AChE and BChE activities in serum of HNSCC patients were significantly lower than in the control group (1.09 ± 0.07 mU/ml for AChE and 176.43 ± 9.05 mU/ml for BChE; p < 0.001 and p = 0.007, respectively). These results are in agreement with published data demonstrating lower ChE levels in serum from cancer patients [30–33].

### Survival and cholinesterase activity

**Fig. 1:**
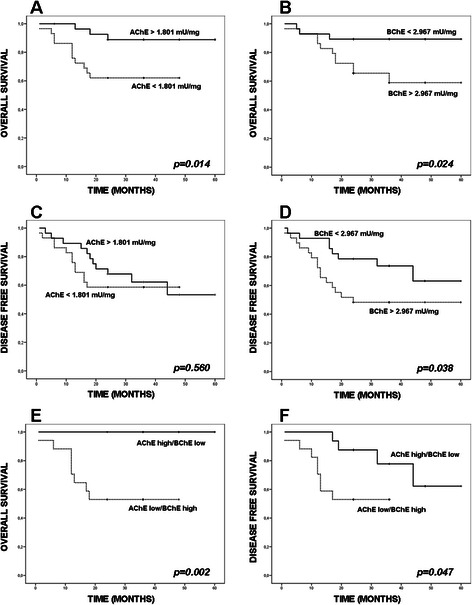
Kaplan-Meier estimated overall (OS) and disease-free survival (DFS) rates according to ChE activity values. Tumours (n = 57) were split into those that exhibited higher or lower values than the 50^th^ percentile for AChE activity (1.801 mU/mg protein; **a**, **c**), for BChE activity (2.967 mU/mg tissue; **b**, **d**) or for the combination of AChE and BChE for OS (**e**) and DFS (**f**). Low tumour AChE activity was found to be statistically associated with adverse OS rate (p = 0.014) (**a**), but not with shorter DFS rate (p = 0.560) (**c**). High BChE activity in tumours was associated with both adverse OS (p = 0.020, **b**) and DFS (p = 0.030; **d**). The combination of AChE and BChE (“AChE high/BChE low” vs “AChE low/BChE high”) were statistically linked with adverse OS (p = 0.002) (**e**) and DFS (p = 0.047) (**f**)

In our attempts to test whether ChE activity might be used as a prognostic marker, overall (OS) and disease-free (DFS) survival rates of patients were measured. The median follow-up time was 29.04 months (range 6–60 months), and a cut-off value of the 50^th^ percentile of AChE (1.801 mU/mg protein) and BChE (2.967 mU/mg protein) was set-up when comparing activity values in tumours with OS and DFS rates of the study population. The results indicated that tumours with AChE activity below 1.801 mU/mg were statistically associated with poorer OS (p = 0.014; Fig. 1a), but not with shorter DFS (p = 0.560; Fig. 1c). As for BChE activity, patients with high tumour BChE activity (>2.967 mU/mg protein) had a poorer prognosis relative to both OS (Fig. 1b; p = 0.024) and DFS (Fig. 1d; p = 0.038). When Survival was evaluated by combining AChE and BChE activities, patients with AChE low/BChE high had shorter OS (p = 0.002) and DFS (p = 0.047) when compared with AChE high/BChE low samples (Fig. 1e-f).

Among the available pathological and clinical variables (Table 1), advanced clinical stage (III + IV) and affected lymph nodes (N+) were significantly associated with shorter survival (Additional file 1: Figure S2 C-D). Subsequently, prediction of shorter survival associated with low AChE activity was tested in the multivariate analysis by Cox proportional hazards regression model, adjusting for clinical stage and for spreading to lymph nodes. Results showed that low AChE activity was an independent prognostic marker respect to clinical stage (ExpB 2.48, CI 1.130-5.444, p = 0.021) and lymph node status (ExpB 2.444, CI 1.073-5.570, p = 0.033). For BChE we found also statistical significance when clinical stage (ExpB 3.517, CI 0.978-12.655, p = 0.044) or lymph node status (ExpB 5.385, CI 1.190-24.365, p = 0.029) were tested.

As the differences in AChE activity were observed comparing mean values in ANCT and HNSCC pieces, OS rates were compared with the ratios of AChE activity in HNSCC and its ANCT sample. Despite the early mortality showed by patients with high difference in AChE activity between ANCT and HNSCC pieces (ratio > 1.92), the results did not reach statistical significance (Additional file 1: Figure S1A; p = 0.071). When the ANCT to HNSCC BChE activity ratio was compared, OS rates also failed in reaching statistical significance (Additional file 1: Figure S1B; p = 0.099).

### Gene expression of cholinergic components in head and neck carcinoma

**Fig. 2:**
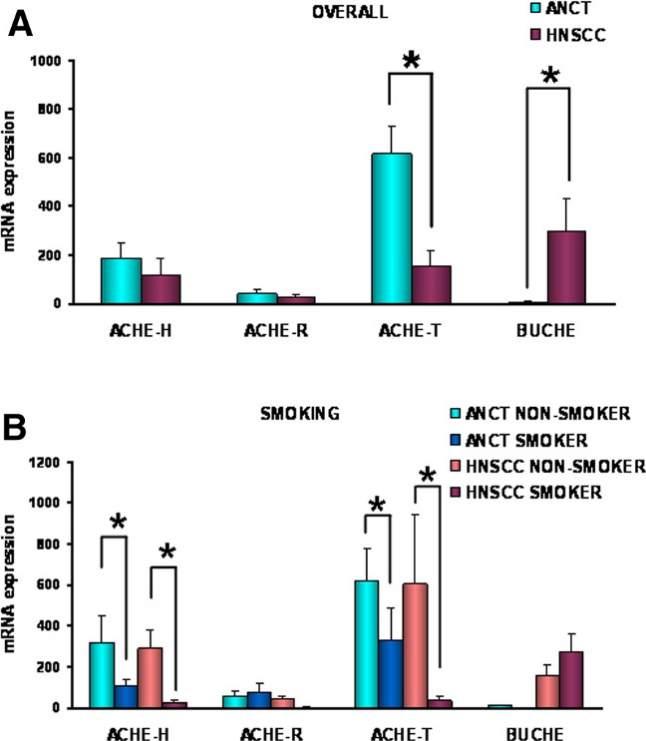
Histograms showing differences between unaffected and cancerous pieces in the levels of the distinct AChE mRNA variants and the single BChE transcript. mRNA of adjacent non cancerous tissues (ANCT; blue bars) and of head and neck squamous cell carcinomas (HNSCC; red bars). mRNA was extracted, retro-transcribed and amplified using the primers indicated in Additional file 1: Table S1. β-actin and GAPDH mRNAs were used as the housekeeping markers. AChE-T, AChE-H, and AChE-R stand for tailed (synaptic) AChE, hydrophobic (erythrocytic) AChE, and read-through AChE. AChE and BChE mRNA levels in HNSCC and ANCT pieces (**a**) and in tissues from smoker and non smoker patients (**b**). Note the lower AChE-T mRNA level (p = 0.036) and higher BChE mRNA level (p = 0.015) in HNSCC than ANCT pieces (**a**) and the significant decrease of both AChE-T and AChE-H mRNA levels in ANCT (dark blue) and HNSCC (dark red) tissues from smoker patients. (* p < 0.05)

The lower ChE activity in HNSCC pieces may arise from gene down-regulation, a possibility that was assessed by measuring AChE and BChE mRNA levels in unaffected and cancerous pieces. The data showed that ANCT samples contained principal AChE-T mRNA and less AChE-H and AChE-R mRNAs and copies). AChE-T mRNA level was significantly lower in HNSCC pieces (Fig. 2a) meanwhile AChE-H and AChE-R mRNAs tended to decrease with cancer but did not reach statistical significance. Contrary to AChE, BChE was found to be up-regulated in head and neck carcinoma as judged by the negligible level of BChE mRNA in ANCT and its strong increase in HNSCC (Fig. 2a).

Since smoking is strongly associated with head and neck cancer, correlation between tobacco exposure and level of expression of AChE and BChE mRNAs was assessed (Fig. 2b). Both ANCT and HNSCC tissues from smoker patients showed much lower level of AChE-H and AChE-T mRNA respect to tissues from non-smoker, indicating that tobacco components downregulate expression of ACh-hydrolyzing enzymes.

Afterwards, considering that the expression of AChE and BChE genes frequently changes with development and/or proliferation states [34], the possibility remained that tumours with distinct differentiation grading displayed unequal patterns of AChE or BChE mRNAs. PCR data indicated that while well and moderately differentiated tumours contained lower AChE mRNA (Additional file 1: Figure S2A), the opposite applied for poorly differentiated tumours (Additional file 1: Figure S2B). Meanwhile, BChE expression was up-regulated regardless of the tumour histological grade (Additional file 1: Figure S2A-B). A comparison of the changing levels of AChE and BChE mRNAs in carcinomas according to their anatomical location also showed differences (Additional file 1: Figure S2C-D).

**Fig. 3:**
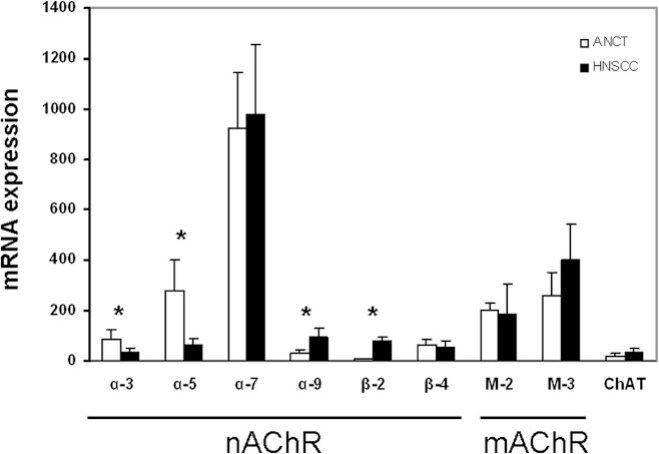
mRNA levels for proteins involved in cholinergic signalling in ANCT (white bars) and HNSCC specimens (black bars). Note the negligible level of ChAT mRNA in ANCT and HNSCC and the absence from the tissue specimens of detectable PRiMA mRNA (not showed). (* p < 0.05)

In our attempts to assess if the expression of the proteins required for establishing a putative ‘oncogenic’ cholinergic system changed with malignancy, the mRNA levels for nAChR and mAChR, ChAT, and PRiMA were also studied. The negligible level of ChAT mRNA in ANCT and the absence of PRiMA mRNA from it, ruled out an active synthesis of ChAT and PRiMA proteins in upper respiratory tract epithelium. However, the observation in ANCT of α7 and α5 mRNAs, and fewer α3, α9, β2 and β4 mRNAs for nAChR subunits (Fig. 3) supported the production in airway epithelium of heteromeric nAChR, consisting of α3, α5, β2 and β4 subunits, and homomeric nAChR made of α7 or α9 subunits. Moreover, the presence of mRNAs for M2 and M3 mAChR in unaffected and cancerous tissues (Fig. 3) supported the translation and membrane targeting of Gi-coupled and Gq-coupled mAChR in airway epithelium. The differences between ANCT and HNSCC in the relative content of the mRNAs for α3, α5, α9, and β2 proteins (Fig. 3) supported cancer-induced changes in the availability of proteins involved in cholinergic signalling.

### Molecular distribution of AChE and BChE in upper tract respiratory epithelium

**Fig. 4:**
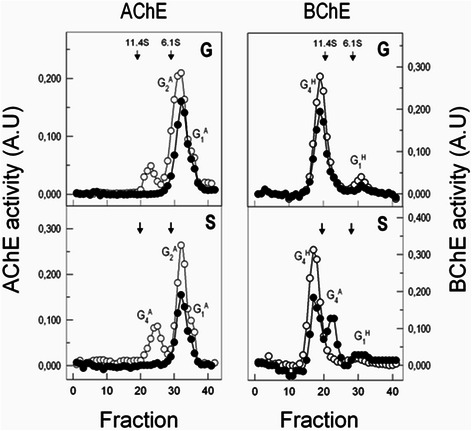
Sedimentation profiles depicting AChE and BChE components in human upper respiratory epithelium. Molecular forms AChE and BChE in unaffected (empty circles) and cancerous tissues (filled circles) in glottis (G) and supraglottis areas (S)

The fact that the normal distribution of ChE components was frequently altered in pathological tissues prompted us to compare the pattern of AChE and BChE molecules in upper tract respiratory epithelium. Sedimentation analysis revealed abundant 4.4 ± 0.2S AChE forms, and fewer 9.7 ± 0.2S and 3.0 ± 0.1S components in ANCT and HNSCC pieces (Fig. 4). According to previous data [25], the major 4.4S forms were assigned to amphiphilic AChE dimers (G_2_^A^), most probably consisting of GPI-linked AChE, arising from the AChE-H mRNA. The 3.0S forms were attributed to GPI-bound G_1_^A^ AChE, and the 9.7S forms to PRiMA-bound tetramers (G_4_^A^), consisting of four AChE-T subunits bonded to PRiMA. The distribution of AChE forms was similar in most ANCT pieces tested, except for G_4_^A^ AChE which lacked in 4 out the 14 pieces analyzed. Of note was the absence of PRiMA-bearing tetramers from HNSCC of glottis and supraglottis locations (Fig. 4).

As regards BChE, sedimentation profiles showed major hydrophilic tetramers (G_4_^H^; 12.0 ± 0.2 S) and less PRiMA-linked tetramers (G_4_^A^; 9.9 ± 0.3 S) and hydrophilic monomers (G_1_^H^; 4.6 ± 0.2 S) in ANCT samples. The presence in airway epithelium of G_1_^H^ BChE (Fig. 4), which lacked from human plasma demonstrated the epithelial cell origin of BChE. Moreover, the neural origin of PRiMA [35] supported a nerve source of the PRiMA-linked BChE tetramers identified in most cancerous pieces of supraglottis location.

### Detection of proteins by western-blotting and activity-based protein profile

**Fig. 5:**
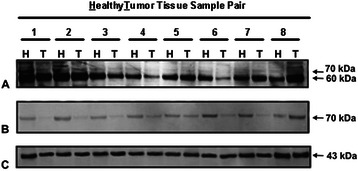
Immunoblotting of AChE in human upper airway epithelium. Proteins in non cancerous (Healthy; H) and cancerous (Tumour, T) pieces were separated by reducing SDS-PAGE and detected by western blotting with anti-AChE antibodies. The use of N19 antibodies allowed us to observe two-three deeply labelled protein bands of about 60-kDa and various fainter bands of 70–76 kDa (**a**). The active site-directed probe Ph-F was able to label the 70–76 kDa bands, but not the 60-kDa proteins (**b**). Accordingly, the deep 60-kDa protein bands in ANCT and HNSCC specimens were assigned to non-catalytic AChE proteins, and the faint 70–76 kDa bands to catalytic proteins. Note the much weaker signal corresponding to catalytic AChE in tumours (T) than healthy (H) tissues. The loaded control was β-actin (**c**)

After reporting that cultured lung cancer cells and tissues possess the capacity to express both catalytically competent and non-competent AChE molecules [8] insights into possible differences in size and abundance of active and inactive AChE subunits were gained by western-blotting of unaffected and cancerous tissue extracts using N19 antibodies. Deep 60-kDa and weak 70–76 kDa protein bands were observed (Fig. 5a), and like in lung epithelia [8], the lack of correlation between the AChE units loaded in the gel lanes and the labelling intensity demonstrated the presence of inactive AChE molecules in ANCT.

Moreover, the usefulness of probes directed to the catalytic site of enzymes (activity-based protein profile; ABPP methodology) to label catalytically competent enzymes in complex proteomes [36] prompted us to take advantage of the efficient labelling of catalytic AChE using Ph-F and of its inability to mark non-catalytic AChE. Sample extracts showed Ph-F labelled 70–76 kDa proteins, whose intensity was fainter in HNSCC than ANCT pieces (Fig. 5b) despite the similar labelling of the 43-kDa β-actin band (Fig. 5c). The fact that the 60-kDa protein failed in being marked by Ph-F supported its assignment to catalytically inactive AChE.

## Discussion

The results reported here demonstrated that human upper airway truck epithelium possesses the capacity to express a wide range of ACh-related proteins, such as AChE, BChE, nAChR, mAChR, and possibly ChAT. These and previous observations [4] lent strong support to the presence in airway epithelium (and possibly in other epithelia) of a physiologically active non-neuronal cholinergic system. This system is expected to be crucial for precise and reliable control of the intensity and duration of cholinergic inputs and down-stream events, including cell growth and proliferation. In spite of the abundant information on cancer-associated changes in ChE activity, assembly of ChE subunits, and processing of their linked oligoglycans in human breast, lymph node, gut, lung, prostate and kidney [8, 19, 37], conclusive proofs of a causal relationship of tumour aggressiveness with ChE changes are still lacking. Nevertheless, several observations have lent weight to a possible role of ChEs in tumorogenesis and tumour biology. These include: 1) the relationship of human astrocytoma aggressiveness and altered patterns of splice-derived AChE variants [34]; 2) the increased labelling of cytoplasm-residing AChE in ovarian cancer [38]; 3) the shorter DFS and OS rates of patients carrying low AChE activity-exhibiting hepatocarcinoma [23]; and 4) the low ChE activity assayed in specimens of advanced prostate cancer [39].

The significant and comparable levels of AChE and BChE activities in ANCT (Table 1) demonstrated that upper respiratory tract was able to regulate the available ACh and, therefore, cholinergic signalling. Moreover, the drop of AChE and BChE activities in head and neck cancers and the differences between ANCT and HNSCC pieces in AChE, BChE, nAChR and mAChR mRNA levels lent strong support to the notion that a non-neuronal cholinergic system may be involved in head and neck malignancy. The heretofore unnoticed observation that patients carrying HNSCC with AChE activity below the cut-off value have a poorer survival rate (Fig. 1) reminds very much the case of hepatocellular carcinoma patients, for which a low level of AChE activity was found to be correlated with an increased risk of post-operative recurrence [23]. These results strengthened the idea of a relationship between a low ACh-hydrolyzing activity and tumour growth in both head and neck carcinoma, and liver carcinoma at least. The potent anti-tumour action that an AChE-loaded adenoviral vector exerts on gastric cancer cells [40] lends support to the tumour growth-promoting facet of ACh.

On the other hand, the well-known capacity of ACh for blocking NFκB production [41, 42] makes it possible that the increased ACh level, arising from the decreased AChE activity in HNSCC and hepatic carcinoma, tightens the blockade of cytokines production, which may provide a tentative explanation for the poor survival prospects of the patients afflicted of HNSCC with low AChE activity. In addition, microRNA regulation may offer key answers to the varying levels of ChE activity in cancerous tissues. The observed changes in the mRNA levels of the tested cholinergic proteins (Figs. 2 and 3) may reflect, in addition to transcriptional differences, changes in micro-RNA regulation [43–45]. In this regards, it is worth mentioning the reported tumour-suppressor activity that synaptic AChE (targeted by miRNA-212) plays in non-small cell lung cancer [46]. This anti-tumour action not only provides a clear proof of the involvement of AChE in tumorigenesis, but it also confirms the participation of miRNA in the control of AChE activity levels (and ACh availability), which encourages researchers to work in the novel and promising field of microRNA-AChE regulation.

In addition, the results of Table 1 suggest a probable causal relationship between cancer-promoting habits (smoking or alcohol intake) and bad tumour prognosis features, such as a poor differentiation stage. The results of Table 1 also make possible that the statistical changes in AChE activity levels between ANCT and HNSCC were related with advanced tumour stages. In conclusion, our results support the notion that the lower the AChE activity in HNSCC, the greater the chance of a poor prognosis, possibly owing to cholinergic over-activation arising from an increased level of ACh in the neighbourhood of cancerous cells. The fact that the differences in AChE activity levels between HNSCC pieces failed in reaching statistical significance with respect to well-accepted pathological features (Table 1) suggested that the decrease of AChE activity may represent an early step in malignant transformation. Nevertheless, the possibility remains that the lower AChE activity in HNSCC represents a specific feature of the cell type from which the tumour emanates.

Contrary to AChE activity, BChE activity above the median value was found associated with bad OS and DFS rates (Table 1, and Fig. 1b, d). This result was unexpected considering the ACh-hydrolyzing capacity of both BChE and AChE, the former working less efficiently. BChE has attracted much attention due to its capacity to hydrolyze cocaine (and other toxic esters) and its ability to scavenge nerve agents and organophosphorous pesticides [47]. Apart from this scavenging action, there is evidence that BChE intervenes in the regulation of intrinsic inflammation and activity of cholinoceptive glial cells, whose appropriate activation and maintenance seem to provide profitable responses [48, 49]. In addition, the repeatedly observed drop of plasma BChE activity in conditions of acute surgical and clinical illness, which lead to the so-called acute phase response [50] may explain the better prospects of patients carrying HNSCC with BChE activity below the cut-off value.

The longer survival of patients carrying HNSCC with higher AChE and lower BChE (Fig. 1e-f) agreed with the findings of others for post-stroke patients [51], whose survival pace was related with higher serum AChE activity and lower BChE activity, and differed from the prospects of patients who underwent cardiovascular events, whose survival was found associated with higher serum AChE and BChE activities [52]. Moreover, the possibility remains that the increased average value for BChE activity in HNSCC reflects compensatory mechanisms to overcome temporal (or permanent) deficiency (or loss) of AChE [53]. If this were the case, an inverse correlation between the decrease of AChE activity in cancerous samples and the increase in BChE activity in them should be expected. In support of the above idea there is the fact that HNSCC exhibits decreased levels of the principal AChE-T and AChE-H mRNAs and increased levels of the BChE mRNA (Fig. 2). However, the response of cells to the AChE deficiency seems to be incomplete as showed the decreased AChE and BChE activities in HNSCC.

The presence in unaffected and malign respiratory tract epithelia of principal amphiphilic AChE dimers (G_2_^A^), most likely consisting of GPI-linked AChE (Fig. 4), agreed with the molecular profiles observed in studies of unaffected and cancerous breast and other epithelial tissues [8, 25, 54]. The predominance in epithelial tissues and blood cells of GPI-linked G_2_^A^ AChE, which arises from the AChE-H mRNA, reinforces the idea that the AChE-H mRNA variant is the principal source (if not the only one) of AChE activity in the airway epithelium and other non-nervous tissues [55]. The presence in most ANCT specimens of nerve-born PRiMA-linked AChE tetramers (Fig. 4), their lack from glottis- and supraglottis cancerous pieces, and the presence in them of PRiMA-linked BChE tetramers agreed with previous histological observations showing tumour-associated remodelling and loss of nerve terminals along head and neck cancer development and motor nerve invasion [56].

Interestingly, the AChE gene expression was found to vary in HSNCC according to their differentiation grade (Additional file 1: Figure S2A-B). So, whilst poorly differentiated tumours were able to maintain or even increase the levels of the three AChE mRNA types, all of them were found decreased in well or moderately differentiated tumours. The increased AChE mRNA level in poorly differentiated HSNCC may reflect the cell attempts to increase catalytic AChE as a means to attenuate cholinergic over-activation. Unfortunately, the attempts seem to be unsuccessful given the lower AChE activity in cancerous than non-cancerous pieces (Table 1). In addition, the decreased AChE-T mRNA level in glottis-located tumours and its increased level in supraglottis tumours (Fig. 2) made unlikely AChE-T mRNA as the leading transcript for AChE activity in unaffected and cancerous pieces. Instead, the decreased AChE-H mRNA levels in glottis and supraglottis tumours strongly supported the proposal of AChE-H mRNA as the principal source of the enzyme activity in non-neural peripheral tissues [55]. Moreover, the opposite changes in AChE-T mRNA levels of glottis and supraglottis tumours suggest that the particular environment surrounding tumour cells may determine transcriptional and post-transcriptional events and, in the case of HNSCC at least, without affecting the AChE gene splicing pattern. Of note is the paradox of an enhanced AChE-T mRNA level in supraglottis tumours and a lower AChE activity in them (Table 1). Post-translational events, including conversion of catalytically incompetent into competent subunits, oligomerization, and rapid secretion of AChE-T made oligomers may explain the lack of correlation between the discrepant levels of AChE-T mRNA and of AChE hydrolyzing activity.

As a whole, the results reported herein unambiguously demonstrate that AChE gene is down-regulated in HNSCC. In support of this statement are the decreased levels for AChE-T, AChE-H and ACh-R mRNAs (Fig. 2A), the weaker labelling of 70–76 kDa catalytic subunits in cancerous pieces, and the fainter signal for non-catalytic 60-kDa subunits in tumours pieces in pairs of ANCT and HNSCC samples (Fig. 5). As for BChE, the decreased enzyme activity (Table 1) in head and neck carcinomas contrasted with the increased mRNA levels in them. This paradox might arise among other causes from decreased translation efficiency, shorter half-life of BChE protein in tumours, or faster release of secretion-destined BChE tetramers.

The identification in ANCT of mRNAs for nAChR, mAChR, and ChEs (Fig. 3), and their changing levels in HNSCC indicate that human upper aero-digestive tract epithelium can produce protein components of a non-neuronal cholinergic system. The observation in ANCT and HNSCC of mRNAs for α7, α5, α3, and α9 AChR subunits (Fig. 3) might lend support to the Hainaut's group proposal, according to which homomeric α7 receptors mediate in proliferative effects and heteromeric α3(β2/β4)α5 receptors in negative inputs [57]. Of note is the widely accepted consideration of α7 receptor as a key mediator of pathological effects in airways of tobacco components [58]. The association of α7 nAChR with breast cancer [59] and the risen α7 mRNA levels in HNSCC support the possibility that α7 nAChR is a target for head and neck cancer.

The first hint supporting a causal relationship of cholinergic activation with tumour growth came from studies showing the presence of AChR in cervical cancer [60], colon cancer [61], non-small cell lung cancer [62, 63] and small cell lung cancer SCLC [64, 65]. Another line of evidence stems from studies showing that impaired cholinergic activity due to abnormal AChR functioning stimulates tumour growth by promoting several hallmarks of cancer cells [66, 67]. Interestingly, the differences in airway epithelium between smokers and non-smokers in nAChR [10] and ACh-hydrolyzing enzymes (Fig. 2B) gene expression firmly support the possibility that the tobacco components are responsible for the changed expression. Thus, it is tempting to speculate that daily and persistent nicotine/nitrosamine exposure may lead to changes in the expression level of the protein components that form a pro-tumorogenic cholinergic system in airway epithelium characterized by an upregulated, pro-proliferative homomeric α7 nicotinic receptors and a downregulation of “inhibitory” nAChR and ACh-hydrolyzing enzymes. The feeding of the system by endogenous ACh would explain the high risk of tumorogenesis that former smoker show after smoking cessation for long time. The combination of increased nicotine-induced ACh synthesis and decreased degradation due to down-expressed ChEs can result in an increase in available ACh in the tumour and to proliferative stimuli to both ‘tumoral’ mAChR and nAChR.

Summarizing, the fact that HNSCC-expressing low AChE activity exhibit a poor prognosis raises the possibility of using this low activity as a reliable prognosis predictor. The increased ACh level, owing to the loss of ACh-hydrolysing activity may lead to over-activation of ACh receptors. So, cholinergic signalling and down-stream events may be exacerbated in HNSCC and by this means favour tumour growth and/or promote a more aggressive tumor phenotype. This study lends strong support regarding the potential use of AChE as a predictor of clinical outcomes, which, eventually, may lead to novel strategies in drug design.

## Conclusion

This study compared ACh-hydrolyzing activity and expression of ACh-related proteins in human upper airway truck epithelium, and found that HNSCC-expressing low AChE activity exhibit a poor prognosis and shorter overall survival. Exacerbated cholinergic signalling and down-stream events may favour tumour growth and/or promote a more aggressive tumor phenotype. This study lends strong support regarding the potential use of AChE as a predictor of clinical outcomes, which, eventually, may lead to novel strategies in drug design.

## Additional file

Additional file 1: **Material and Methods.** Primers for relative quantitative PCR. **Table S1.** List of pairs of primers designed for relative quantitative PCR. **Figure S1.** Kaplan-Meier estimated overall survival according to the ratio ANCT to HNSCC ChE activity values (**A, B**) and to clinical variables as lymph nodes (**C**) and clinical staging (**D**). Tumours (n=57) were split into those that exhibited higher or lower values than the 50^th^ percentile for the ratios of unaffected to cancerous AChE (**A**) and BChE (**B**). Statistically significant shorter survival was found in patients with lymph node affected (**C**) and advanced clinical stage (**D**). **Figure S2.** Histograms showing differences between unaffected and cancerous pieces in the levels of the distinct AChE mRNA variants and the single BChE transcript. AChE and BChE mRNA levels in HNSCC and ANCT pieces in well or moderately differentiated (**A**) and poorly differentiated tumours **(B)**; and in tumours located in glottis (**C**) or supraglottis areas **(D)**. Note the opposite changes in AChE-T mRNA levels in poorly differentiated (**A**) and well differentiated (**B**) tumours and the anatomical location-associated changes in AChE mRNAs levels. (* p< 0.05).

## Abbreviations

HNSCC: Head and neck squamous cell carcinoma
ANCT: Adjacent non-cancerous tissue
ACh: Acetylcholine
AChE: Acetylcholinesterase
BChE: Butyrylcholinesterase
nAChR: nicotinic acetylcholine receptor
mAChR: muscarinic acetylcholine receptor
ChAT: Choline acetyl-transferase
