# Supplementary material for: Dysregulated cholinergic network as a novel biomarker of poor prognostic in patients with head and neck squamous cell carcinoma
Source: BMC Cancer. 2015 May 10;15:385. doi: 10.1186/s12885-015-1402-y (PMC4435806; doi:10.1186/s12885-015-1402-y)
Supplement: Additional file 1: — Material and Methods. Primers for relative quantitative PCR. Table S1. List of pairs of primers designed for relative quantitative PCR. Figure S1. Kaplan-Meier estimated overall survival according to the ratio ANCT to HNSCC ChE activity values (A, B) and to clinical variables as lymph nodes (C) and clinical staging (D). Tumours (n=57) were split into those that exhibited higher or lower values than the 50th percentile for the ratios of unaffected to cancerous AChE (A) and BChE (B). Statistically significant shorter survival was found in patients with lymph node affected (C) and advanced clinical stage (D). Figure S2. Histograms showing differences between unaffected and cancerous pieces in the levels of the distinct AChE mRNA variants and the single BChE transcript. AChE and BChE mRNA levels in HNSCC and ANCT pieces in well or moderately differentiated (A) and poorly differentiated tumours (B); and in tumours located in glottis (C) or supraglottis areas (D). Note the opposite changes in AChE-T mRNA levels in poorly differentiated (A) and well differentiated (B) tumours and the anatomical location-associated changes in AChE mRNAs levels. (* p< 0.05). [file 12885_2015_1402_MOESM1_ESM.doc]

**Additional file 1**

| **Table S1. List of pairs of primers designed for relative quantitative PCR.** | | |
| --- | --- | --- |
| ***Gene*** | **Primer sequences** | **Length (bp)** |
| *AChE* variant T | Forward 5´-AACTTTGCCCGCACAGGGGA-3´ | 203 |
|  | Reverse 5´-GCCTCGTCGAGCGTGTCGGT-3´ |
| *AChE* variant H | Forward 5´-AACTTTGCCCGCACAGGGGA-3´ | 201 |
|  | Reverse 5´-GGGAGCCTCCGAGGCGGT-3´ |
| *AChE* variant R | Forward 5´-CCCCTGGACCCCTCTCGAAAC-3´ | 315 |
|  | Reverse 5´-ACCTGGCGGGCTCCCACTC-3´ |
| *BChE* | Forward 5´-TGCAAAATATGGGAATCCAAA-3´ | 208 |
|  | Reverse 5´-CCACTCCCATTCTGCTTCAT-3´ |
| *ChAT* | Forward 5´-AGCCTGCTGCAATCAGTTCT-3´ | 202 |
|  | Reverse 5´-GGTGGAGTCTTTCACGAGGA-3´ |
| *PRiMA* | Forward 5´-CCTGCTCCAAAGTGACTGAC-3´ | 250 |
|  | Reverse 5´ -GAGTCAGCTGAGGCTGGTCT-3´ |
| *Nicotinic α-3* | Forward 5´-TGTCTCAGCTGGTGAAGGTG-3´ | 191 |
|  | Reverse 5´-GAAATCCCCAACAGCATTGT -3´ |
| *Nicotinic α-5* | Forward 5´-TGTCCGTGAGGTTGTTGAAG-3´ | 163 |
|  | Reverse 5´-TGCATTTCCAATATGAACTGGT-3´ |
| *Nicotinic α-7* | Forward 5´-GGACGTGGATGAGAAGAACC-3´ | 126 |
|  | Reverse 5´-CCATCTGGGAAACGAACAGT-3´ |
| *Nicotinic α-9* | Forward 5´-GACTGAGAGCTGCAGAGACG-3´ | 197 |
|  | Reverse 5´-GCGGATCCACAAATAAGCAG-3´ |
| *Nicotinic β-2* | Forward 5´-GATGACCAGAGCGTGAGTGA-3´ | 142 |
|  | Reverse 5´-TGGTGGTGGTGTAGTTCTGG -3´ |
| *Nicotinic β-4* | Forward 5´-TCCCTGGTCCTTTTCTTCCT-3´ | 199 |
|  | Reverse 5´-CTCGCTCATTCACGCTGATA-3´ |
| *Muscarinic 2* | Forward 5´-AAGACCCCGTTTCTCCAAGT-3´ | 199 |
|  | Reverse 5´-GAGGCAACAGCACTGACTGA -3´ |
| *Muscarinic 3* | Forward 5´-CCTTCAAGGAAGCCACTCTG-3´ | 197 |
|  | Reverse 5´-GGGTATGCAGCTGTCACAAA -3´ |
| *β-actin* | Forward 5´-AGAAAATCTGGCACCACACC-3´ | 143 |
|  | Reverse 5´-GGGGTGTTGAAGGTCTCAAA-3´ |
| *GAPDH* | Forward 5´-ATGGGGAAGGTGAAGGTCG-3´ | 107 |
|  | Reverse 5´-GGGTCATTGATGGCAACAATATC-3´ |

Material and Methods

Primers for relative quantitative PCR

**Figure S1**

**
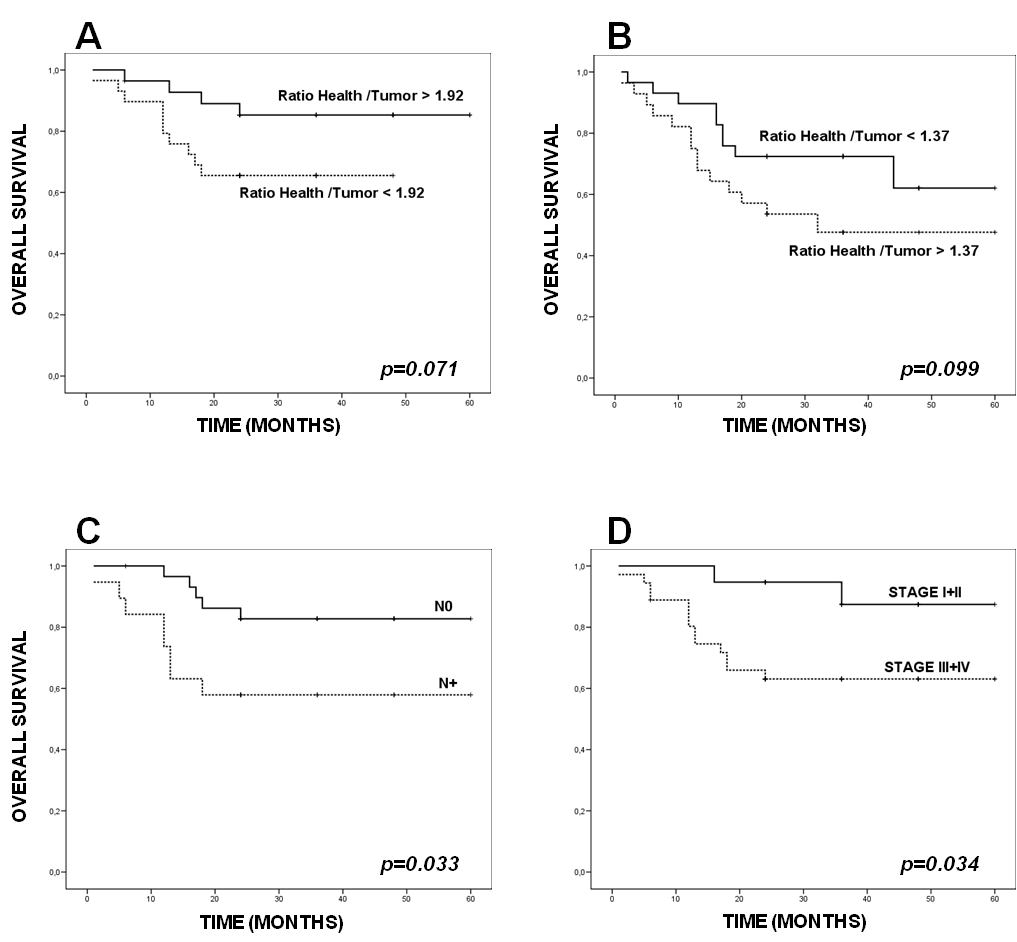
**

**Figure S1.** Kaplan-Meier estimated overall survival according to the ratio ANCT to HNSCC ChE activity values (**A, B**) and to clinical variables as lymph nodes (**C**) and clinical staging (**D**). Tumours (n=57) were split into those that exhibited higher or lower values than the 50^th^ percentile for the ratios of unaffected to cancerous AChE (**A**) and BChE (**B**). Statistically significant shorter survival was found in patients with lymph node affected (**C**) and advanced clinical stage (**D**).

**Figure S2**

**
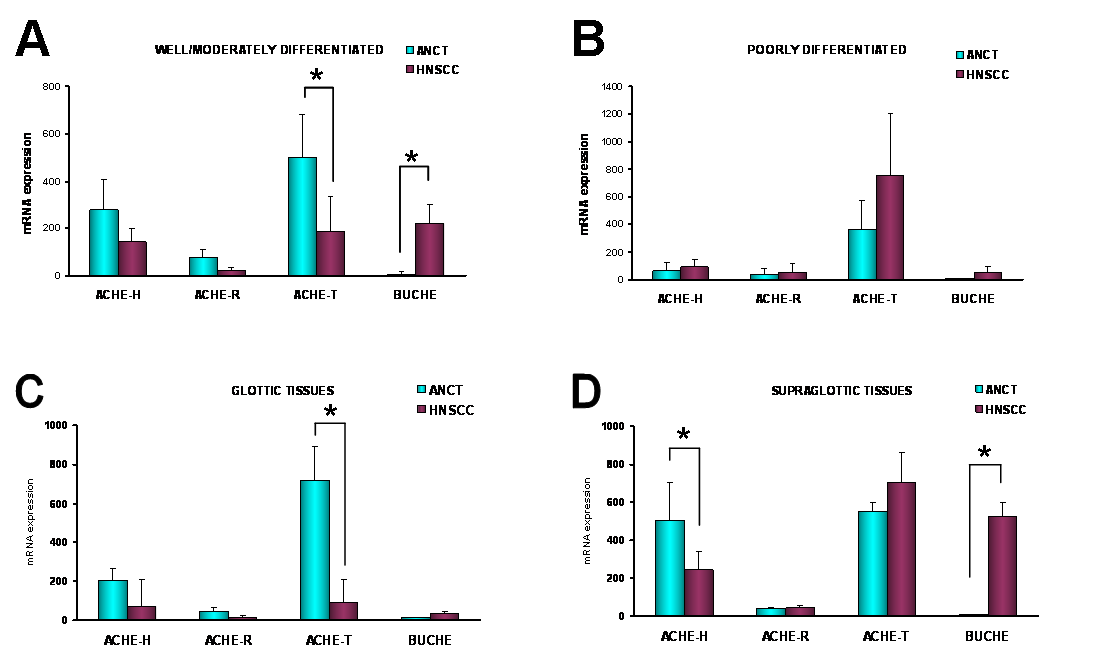
**

**Figure S2.** Histograms showing differences between unaffected and cancerous pieces in the levels of the distinct AChE mRNA variants and the single BChE transcript. AChE and BChE mRNA levels in HNSCC and ANCT pieces in well or moderately differentiated (**A**) and poorly differentiated tumours **(B)**; and in tumours located in glottis (**C**) or supraglottis areas **(D)**. Note the opposite changes in AChE-T mRNA levels in poorly differentiated (**A**) and well differentiated (**B**) tumours and the anatomical location-associated changes in AChE mRNAs levels. (* p< 0.05).
